# Assessment of Antibody and T-Cell Responses to the SARS-CoV-2 Virus and Omicron Variant in Unvaccinated Individuals Recovered From COVID-19 Infection in Wuhan, China

**DOI:** 10.1001/jamanetworkopen.2022.9199

**Published:** 2022-04-27

**Authors:** Li Guo, Qiao Zhang, Chongyang Zhang, Tingxuan Huang, Lili Ren, Bin Cao, Jianwei Wang

**Affiliations:** 1 National Health Commission Key Laboratory of Systems Biology of Pathogens and Christophe Mérieux Laboratory, Institute of Pathogen Biology, Chinese Academy of Medical Sciences and Peking Union Medical College, Beijing, China; 2 Department of Respiratory and Critical Care Medicine, West China Hospital, Sichuan University, Chengdu, Sichuan, China; 3 Department of Pulmonary and Critical Care Medicine, National Center for Respiratory Medicine, Center of Respiratory Medicine, National Clinical Research Center for Respiratory Diseases, China-Japan Friendship Hospital, Beijing, China; 4 Institute of Respiratory Medicine, Chinese Academy of Medical Science, Beijing, China

## Abstract

This cohort study examines immune system responses to the Omicron strain of the SARS-CoV-2 virus among unvaccinated individuals in Wuhan, China, who recovered from infection with the initial strain of the virus.

## Introduction

The SARS-CoV-2 Omicron variant, which harbored 32 mutations in spike glycoproteins (S),^1^ raised concern over the virus escaping from immunity induced by vaccination or natural infection.^2,3^ However, the full extent to which the Omicron variant evades existing vaccine- or infection-derived antibodies, especially memory T-cell responses, has not been well characterized. In this cohort study, we assessed the antibody and T-cell responses to SARS-CoV-2 Wuhan and Omicron strains in individuals recovered from COVID-19.

## Methods

Participants were patients who recovered from COVID-19 and were discharged from the Wuhan Research Center for Communicable Disease Diagnosis and Treatment at the Chinese Academy of Medical Sciences between January 7 and May 29, 2020. Participants were recruited as part of a longitudinal study between December 16, 2020, and January 27, 2021, and did not receive SARS-CoV-2 vaccine.^4^ The study was approved by the institutional review boards of Wuhan Research Center for Communicable Disease Diagnosis and Treatment and the Chinese Academy of Medical Sciences. Written informed consent was obtained from each patient. This study followed the Strengthening the Reporting of Observational Studies in Epidemiology (STROBE) reporting guideline.

Immunoglobulin G (IgG) response against Wuhan strain and Omicron S and receptor binding domain (RBD) were determined by enzyme-linked immunosorbent assay (ELISA) using 113 plasma samples from 113 participants (group 1). Neutralizing antibodies against the Wuhan strain and the Beta, Delta, and Omicron variants were analyzed using pseudovirus neutralization assays using 40 plasma samples from 40 participants (group 2), calculated as 50% inhibitory doses (ID50). SARS-CoV-2–specific memory T-cell responses were accessed using interferon (IFN)-enzyme–linked immunospot (ELISpot) using 41 plasma samples from 41 participants. To investigate T-cell responses, we designed 2 sets of peptide pools: one for the Omicron-mutated regions of the S, N, M, and E proteins and the other for the Wuhan strain–containing homologous peptides. Comparison of IgG seropositivity was done with a χ^2^ test. Multiple comparisons were performed using the Kruskal-Wallis test. Paired comparisons were compared using a 2-tailed Wilcoxon matched-pairs signed-rank test. *P* < .05 was considered to be statistically significant in 2-tailed tests. All statistical analysis was conducted using Prism version 9.3.0 (GraphPad). Additional details of study methods are provided in the Supplement.

## Results

This study included 113 adults; median (IQR) age was 57 (48-65) years, and 73 (64.6%) were men (Table). Seropositivity of Omicron S–IgG and RBD-IgG was significantly lower than those of Wuhan strain (Omicron S-IgG: 80.5% vs 97.3%; *P* < .001; RBD-IgG: 43.4% vs 97.3%; *P* < .001) (Figure). Plasma samples had significantly reduced binding capacity for Omicron S and RBD when compared with those of Wuhan strain (median [IQR] titers: S-IgG, 0.37 [0.28-0.50] vs 0.61 [0.48-0.78]; RBD-IgG, 0.19 [0.16-0.22] vs 0.33 [0.27-0.41]) (Figure).

**Table.** Demographic Features of Patients Who Recovered From COVID-19

Characteristics	Total	Moderate	Severe	Critical
**Group 1**				
No. of participants	113	37	43	33
Age, median (IQR), y	57 (48-65)	63 (50-68)	54 (42-64)	53 (46-62)
Sex, No. (%)				
Men	73 (64.6)	21 (56.8)	31 (72.1)	21 (63.6)
Women	40 (35.4)	16 (43.2)	12 (46.5)	12 (36.4)
Days after infection, median (IQR)	353 (342-360)	343 (335-359)	352 (342-359)	359 (351-369)
**Group 2**				
No. of participants	41	21	20	NA
Age, median (IQR), y	63 (48-69)	61 (49-69)	65 (44-70)	NA
Sex, No. (%)				
Men	17 (41.5)	8 (38.1)	9 (45)	NA
Women	24 (58.5)	13 (61.9)	11 (55)	NA
Days after infection, median (IQR)	365 (353-372)	355 (352-367)	366 (357-380)	NA

Abbreviation: NA, not applicable.

**Figure.**
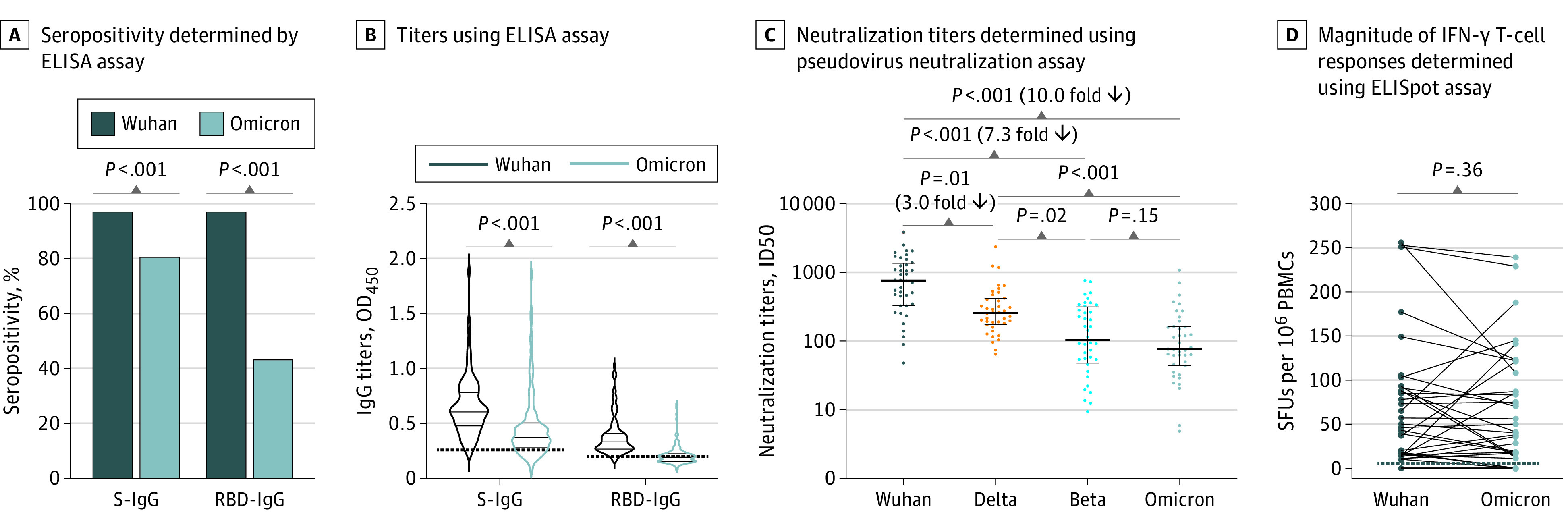
The Antibody and T-Cell Responses to SARS-CoV-2 Wuhan Strain and Omicron Variant ELISA indicates enzyme-linked immunospot; ELISpot, ELI absorbent spot; ID50, 50% inhibitory doses; IFN, interferon; OD, optical density; PBMC, peripheral blood mononuclear cell; RBD, receptor binding domain; S-IgG, spike protein immunoglobulin G; SFU, spot-forming unit. For panel A, 2-tailed *P* values were determined using a *χ^2^* test. In panel B, solid lines in violin denote the median and IQR of antibody titres; dotted lines, the cut-off value. Paired plasma antibody titers were compared using a 2-tailed Wilcoxon matched-pairs signed-rank test. In panel C, lines denote the median and IQR of neutralizing antibody titers. Multiple comparisons of neutralizing antibody titers were performed using the Kruskal-Wallis test followed by a post hoc Dunn correction. In panel D, lines denote the cut-off value of ELISpot assay. Paired T-cell responses were compared using a 2-tailed Wilcoxon matched-pairs signed-rank test.

Neutralizing antibodies against Wuhan strain were detected in each of these individuals (median [IQR] ID50 titers, 764.2 [332.1-1370.0]). However, the median (IQR) ID50 titers were 76.7 (44.0-163.6) for Omicron. Median neutralizing antibody ID50 titers for Beta and Delta variants were 104.5 (47.9-315.5) and 76.7 (44.0-163.6), respectively. The median antibody levels against the Wuhan strain (764.2 [332.1–1370.0]) were significantly higher than those of Delta, Beta, and Omicron variants, whereas the levels for Beta and Omicron were not significantly different (Figure).

The positive rate of T-cell responses to the Wuhan strain and Omicron variant were 78.0% (32 of 41 participants) and 70.7% (29 of 41 participants) (*P* = .45), respectively. IFN-γ responses to the Omicron pool were equal for 21 participants (51.2%), higher for 9 participants (22.0%), and lower for 11 participants (26.8%) than those of the Wuhan strain. However, the overall SARS-CoV-2–specific IFN-γ responses showed no significant differences between Wuhan and Omicron strains (Figure).

## Discussion

In our cohort, the neutralization efficacy of individuals recovered from Wuhan strain without repeated infection and vaccination against Omicron was lower than the original Wuhan strain and Delta variant. Our data and previously studies suggest that Omicron is resistant to vaccine- and infection-elicited antibody responses,^2,3^ but elicits T-cell responses similar to other variants.^5^ Omicron may be less likely to cause severe disease in those who have previously been vaccinated or infected because of T-cell cross-reactivity. A limitation of this study is that the correlations of immune responses in plasma and blood cells with those in respiratory mucosa were not assessed. Continuous surveillance is needed to monitor the incidence of breakthrough infections by Omicron.

## Notes

Supplement.
eMethods.
**eTable 1.** Wuhan Peptide Pool **eTable 2.** Omicron Peptide Pool
