# Supplementary material for: Assessment of Antibody and T-Cell Responses to the SARS-CoV-2 Virus and Omicron Variant in Unvaccinated Individuals Recovered From COVID-19 Infection in Wuhan, China
Source: JAMA Netw Open. 2022 Apr 27;5(4):e229199. doi: 10.1001/jamanetworkopen.2022.9199 (PMC9047635; doi:10.1001/jamanetworkopen.2022.9199)
Supplement: Supplement. — eMethods. eTable 1. Wuhan Peptide Pool eTable 2. Omicron Peptide Pool [file jamanetwopen-e229199-s001.pdf]

Supplemental Online Content

Guo L, Zhang Q, Zhang C, et al. Assessment of antibody and T-cell responses to the SARS-CoV-2 virus and omicron variant in unvaccinated individuals recovered from COVID-19 infection in Wuhan, China. *JAMA Netw Open.* 2022;5(4):e229199. doi:10.1001/jamanetworkopen.2022.9199

eMethods.

eTable 1. Wuhan Peptide Pool

eTable 2. Omicron Peptide Pool

This supplemental material has been provided by the authors to give readers additional information about their work.

© 2022 Guo L et al. *JAMA Network Open.*

Supplement methods

Cohorts

Participants were recovered COVID-19 patients who were discharged from Wuhan Research Center for Communicable Disease Diagnosis and Treatment, Chinese Academy of Medical Sciences between Jan 7 and May 29, 2020. Patients received a follow-up visit between Dec 16, 2020 and Jan 27, 2021. The individual studied here is part of the larger longitudinal cohort study.[1]

The study included two groups. In group one, 113 plasma samples were taken from 113 recovered patients to perform ELISA assay, in which a total of 40 plasmas were further used for pseudovirus neutralization assays. In group two, PBMCs were collected from 41 individuals and analyzed by ELISpot to detect the SARS-CoV-2-specific memory T cell responses.

Inclusion and exclusion criteria

Inclusion criteria:

All patients with laboratory confirmed COVID-19 who were discharged from Wuhan Research Center for Communicable Disease Diagnosis and Treatment, Chinese Academy of Medical Sciences (Wuhan, China) between Jan 7 and May 29, 2020.

Exclusion criteria

(1) those who died before the follow-up visit,

(2) those for whom follow-up would be difficult owing to psychotic disorder, dementia, or re-admission to hospital attributed to underlying diseases,

(3) those who were unable to move freely due to concomitant osteoarthropathy or immobile before or after discharge due to diseases such as stroke or pulmonary embolism,

(4) those who declined to participate,

(5) those unable to be contacted, and

(6) those living outside of Wuhan or in nursing or welfare homes.

Plasma and PMBC isolation

Venous blood was collected from participants and processed within 12 h to isolate plasma and

© 2022 Guo L et al. *JAMA Network Open.*

peripheral blood mononuclear cell (PBMCs). Plasma was separated by centrifugation at 300 x g for 10 minutes and stored at -80°C until testing. PBMCs were isolated from blood using Ficoll-Paque PLUS (GE Healthcare, Chicago, IL) according to the manufacturer's instructions. Isolated PBMCs were frozen in 90% heat-inactivated fetal bovine serum (FBS, Hyclone, Northbrook, IL) supplemented with 10% DMSO (Sigma-Aldrich, St. Louis, MO, USA), and stored in liquid nitrogen before analysis.

Peptide synthesis

Peptides covering the mutated regions of S, N, M, and E protein in Omicron and the corresponding Wuhan strain control pool containing homologous peptides were synthesized (purity >90%; Sangon Biotech, Shanghai, China) (eTable 1, eTable 2).

Enzyme-linked immunosorbent assay (ELISA)

IgG antibody titres against the spike protein (S) and receptor binding domain (RBD) of SARS-CoV-2 Wuhan strain and Omicron strain were evaluated using the enzyme-linked immunosorbent assay (ELISA). Briefly, 20 ng of RBD and S protein of Wuhan strain and Omicron variant (Sino Biological, Beijing, China) were used as coating protein, respectively. Plasma samples were diluted 1/400 with 0.5% bovine serum albumin (BSA) and incubated for 1 h at 37°C. After washing, goat anti-human Fc specific polyclonal IgG (Sigma Aldrich, St Louis, MO, USA) antibodies were added to the plates at a dilution of 1/60 000 with 0.5% BSA. After 1 h of incubation at 37°C, the plates were washed and developed with 100 µL substrate solutions A (3,3',5,5'-tetramethylbenzidine) and B (hydrogen peroxide) in each well (Wantai Biotech Corp, Beijing, China). The reaction was stopped by adding 50 µL of 2 M sulfuric acid. Optical density at 450 nm (OD450) was determined with a multifunctional microplate reader SpectraMax M5 (Molecular Devices, Sunnyvale, CA, USA). Cut-off values were determined by calculating the mean absorbance at 450 nm of negative plasma plus 3-fold SD values, which were 0.20 and 0.20 for Wuhan-RBD- IgG and Omicron-RBD-IgG, 0.26, and 0.26 for Wuhan-S- IgG and Omicron-S-IgG, respectively.

Pseudovirus neutralization assays

© 2022 Guo L et al. *JAMA Network Open.*

Pseudovirus samples of SARS-CoV-2 Wuhan and omicron strains were generated by co-transfection of 293T cells with pLenti-Luciferase, psPAX2 and viral S protein expression plasmids, pCAGGS-WH-S, pCAGGS-Omicron-S. Neutralization assays were performed as follows. Briefly, 293T-ACE2 cells were seeded in a 96-well plate at a concentration of 1×10^4 cells per well in 100 μL of DMEM with 10% FBS, and cultured 12–16 hours. Fivefold serially diluted plasma starting at 1:20 from recovered patients were incubated with SARS-CoV-2 pseudotyped virus for 1h at 37 °C. The mixture was added to 293T-ACE2 cells. After 48h post-infection, luciferase activity was measured with a luciferase assay system (Promega, E1501) on a multifunctional microplate reader SpectraMax M5. The titers of SARS-CoV-2–specific NAbs were calculated as a 50% half-maximal inhibitory does (ID50) and expressed as the dilution of plasma that resulted in a 50% reduction of luciferase luminescence compared with virus control in single-round pseudovirus infection assay.

Ex-vivo ELISpot assay

Peptides containing amino acid mutations and deletions in S, N, M, and E protein of the omicron variant strain and the corresponding Wuhan strain peptides were synthesized (purity >90%; Sangon Biotech, Shanghai, China).

Ex-vivo Interferon-γ (IFN-γ) enzyme-linked immunospot (ELISpot) assays were performed using cryopreserved PBMCs with a Human IFN-γ ELISpot kit (Mabtech, NS, Sweden) following the manufacturer's instructions. Briefly, cryopreserved PBMCs were thawed and washed once with the RPMI-1640 medium containing 10% FBS and rested overnight prior to assay. Human IFN-γ pre-coated plates were washed with phosphate-buffered saline (PBS), and blocked with culture media containing 10% FBS for 30 min. Total 2×10^5 PBMC per well were stimulated in duplicates with overlapping peptide pools at a final concentration of 2 μg/ml for 24 h. DMSO were used as negative controls. Phorbol myristate acetate (PMA)/ionomycin (Multi Science, Hangzhou, China) was used as positive control. Spots were counted using an AID ELISPOT Reader System (AID GmbH, Strasberg, Germany). Mean spots of the negative control wells were subtracted from the test wells to quantify the intensity of antigen-specific T cell responses, and the results were presented as Spot Forming Unit per 10^6 PBMC (s.f.u./10^6 PBMC). T cell responses were considered positive if the mean spot count was ≥ 3-fold higher than the mean

© 2022 Guo L et al. *JAMA Network Open.*

spot of the negative control and ≥10 s.f.u./10^6 PBMCs. If negative control wells had >100 s.f.u. /10^6 PBMC or positive control wells had <1000 s.f.u/10^6 PBMC, the results were excluded from further analysis.

Outcomes

The primary outcomes were NAb titers and T cell responses. The titers of SARS-CoV-2–specific NAbs were expressed as a 50% half-maximal inhibitory does (ID50). T cell responses were expressed as magnitude of IFN-γ produced by SARS-CoV-2 specific-T cells. The secondary outcomes included S-, RBD-IgG, which were expressed as optical density at 450 nm (OD 405); demographic features of recovered patients, including age, sex, and days after infection.

Ethics approval

The study was approved by the Institutional Review Boards of Wuhan Research Center for Communicable Disease Diagnosis and Treatment, Chinese Academy of Medical Sciences (KY-2020-80.01). Written informed consent was obtained from each COVID-19 patient.

Statistical analysis

The comparison of IgG seropositivity was done with χ^2 test. Multiple comparisons of neutralising antibody titers among Wuhan strain and Beta, Delta, and Omicron variants were performed using the Kruskal-Wallis test followed by a post hoc Dunn's correction. Paired plasma antibody titers and T cell responses were compared using a two-tailed Wilcoxon matched-pairs signed-rank test. A two-sided $P < .05$ was considered to be statistically significant. All statistical analysis was conducted using GraphPad Prism 9.3.0 (GraphPad Software, San Diego, CA).

© 2022 Guo L et al. *JAMA Network Open.*

References

1. Huang L, Yao Q, Gu X, et al. 1-year outcomes in hospital survivors with COVID-19: a longitudinal cohort study. *Lancet.* 2021; 398(10302): 747-758. doi: 10.1016/S0140-6736(21)01755-4.

© 2022 Guo L et al. *JAMA Network Open.*

eTable 1. Wuhan Peptide Pool

Protein	Mutation	Amino acid sequence
Spike	A67V, H69del, V70del	FSNVTWFH**A**IHVSGTNGTKR
Spike	T95I	NDGVYFAS**T**EKSNIIRGW
Spike	G142D, V143del, Y144del, Y145del	FQFCNDPFL**GVYY**HKNNKSW
Spike	N211del, L212I, ins214EPE	IYSKHTPI**NL**VRDLPQGF
Spike	G339D	NITNLCPF**G**EVFNATRFA
Spike	S371L, S373P, S375F	DYSVLYN**S**A**S**F**S**TFKCYGVS
Spike	K417N	RQIAPGQTG**K**IADYNYKLPD
Spike	N440K, G446S	VIAWNSN**N**LDSKV**G**GNYNYL
Spike	S477N, T478K, E484A	TEIYQAG**ST**PCNGV**E**GFNCY
Spike	Q493R, G496S, Q498R, N501Y, Y505H	FNCYFPL**Q**SY**G**F**Q**PT**N**GVG**Y**
Spike	Q493R, G496S, Q498R, N501Y, Y505H	**Q**SY**G**F**Q**PT**N**GVG**Y**QPYRVVV
Spike	T547K	VNFNFNGL**T**GTGVLTESN
Spike	D614G	NQVAVLYQ**D**VNCTEVPVA
Spike	H655Y	RAGCLIGAE**H**VNNSYECDI
Spike	N679K, P681H	CASYQTQT**N**S**P**RRARSVA
Spike	N764K	YGSFCTQL**N**RALTGIAVE
Spike	D796Y	IYKTPPIK**D**FGGFNFSQI
Spike	N856K	DLICAQKF**N**GLTVLPPLL
Spike	Q954H	LGKLQDVVN**Q**NAQALNTLVK
Spike	N969K	TLVKQLSS**N**FGAISSVLN
Spike	L981F	ISSVLNDI**L**SRLDKVEAE
NP	P13L	NQRNAPRITFGG**P**SDSTG
NP	E31del, R32del, S33del	STGSNQNG**ERS**GARSKQR
NP	R203K, G204R	SRNSTPGSS**RG**TSPARMA
Envelope	T9I	MYSFVSEE**T**GTLIVNSVL
Membrane	D3G	MA**D**SNGTITVEELKKLL
Membrane	Q19E	ITVEELKKLLE**Q**WNLVI
Membrane	A63T	LWLLWPVTL**A**CFVLAAVY

© 2022 Guo L et al. *JAMA Network Open.*

eTable 2. Omicron Peptide Pool

Protein	Mutation	Amino acid sequence
Spike	A67V, H69del, V70del	FSNVTWFH**V**ISGTNGTKR
Spike	T95I	NDGVYFAS**I**EKSNIIRGW
Spike	G142D, V143del, Y144del, Y145del	FQFCNDPFL**D**HKNNKSW
Spike	N211del, L212I, ins214EPE	IYSKHTPI**I**VR**EPE**DLPQGF
Spike	G339D	NITNLCPF**D**EVFNATRFA
Spike	S371L, S373P, S375F	DYSVLYN**L**A**P**F**F**TFKCYGVS
Spike	K417N	RQIAPGQTG**N**IADYNYKLPD
Spike	N440K, G446S	VIAWNSN**K**LDSKV**S**GNYNYL
Spike	S477N, T478K, E484A	TEIYQAG**NK**PCNGV**A**GFNCY
Spike	Q493R, G496S, Q498R, N501Y, Y505H	FNCYFPL**R**SY**S**F**R**PT**Y**GVG**H**
Spike	Q493R, G496S, Q498R, N501Y, Y505H	**R**SY**S**F**R**PT**Y**GVG**H**QPYRVVV
Spike	T547K	VNFNFNGL**K**GTGVLTESN
Spike	D614G	NQVAVLYQ**G**VNCTEVPVA
Spike	H655Y	RAGCLIGAE**Y**VNNSYECDI
Spike	N679K, P681H	CASYQTQT**K**S**H**RRARSVA
Spike	N764K	QYGSFCTQL**K**RALTGIAV
Spike	D796Y	IYKTPPIK**Y**FGGFNFSQI
Spike	N856K	DLICAQKF**K**GLTVLPPLL
Spike	Q954H	LGKLQDVVN**H**NAQALNTLVK
Spike	N969K	TLVKQLSS**K**FGAISSVLN
Spike	L981F	ISSVLNDI**F**SRLDKVEAE
NP	P13L	NQRNALRITFGG**L**SDSTG
NP	E31del, R32del, S33del	STGSNQNGGARSKQR
NP	R203K, G204R	SRNSTPGSS**KR**TSPARMA
Envelope	T9I	MYSFVSEE**I**GTLIVNSVL
Membrane	D3G	MA**G**SNGTITVEELKKLL
Membrane	Q19E	ITVEELKKLLE**E**WNLVI
Membrane	A63T	LWLLWPVTL**T**CFVLAAVY

© 2022 Guo L et al. *JAMA Network Open.*
